# Salicylic Acid in the Treatment of Lupus

**Published:** 1879-09

**Authors:** 


Salicylic Acid in the Treatment of Lupus.—Dr. A. Melio reports a case of ulcerated lupus of the face, of five years' duration, in which he employed salicylic acid locally, after all the usual applications had proved ineffective. The ulcerating surface was painted three times a day with a solution of six parts salicylic acid in twenty parts glycerine. After a few days the readily bleeding vegetations withered, and the floor of the ulcer took on a healthy appearance ; at the end of the month cicatrization was completed. The patient at the same time took arsenic internally.—*Gazette Degli Ospedali*, and *Med. Chir Rundschau.*

